# Numerical and Experimental Investigation of the Hydrodynamics in the Single-Use Bioreactor Mobius^®^ CellReady 3 L

**DOI:** 10.3390/bioengineering9050206

**Published:** 2022-05-11

**Authors:** Diana Kreitmayer, Srikanth R. Gopireddy, Tomomi Matsuura, Yuichi Aki, Yuta Katayama, Taihei Sawada, Hirofumi Kakihara, Koichi Nonaka, Thomas Profitlich, Nora A. Urbanetz, Eva Gutheil

**Affiliations:** 1 Interdisciplinary Center for Scientific Computing, Heidelberg University, 69120 Heidelberg, Germany; diana.kreitmayer@iwr.uni-heidelberg.de; 2 Pharmaceutical Development, Daiichi-Sankyo Europe GmbH, 85276 Pfaffenhofen an der Ilm, Germany; srikanth.gopireddy@daiichi-sankyo.eu (S.R.G.); thomas.profitlich@daiichi-sankyo.eu (T.P.); nora.urbanetz@daiichi-sankyo.eu (N.A.U.); 3 Biologics Technology Research Laboratories, Biologics Division, Daiichi-Sankyo Co., Ltd., Ohra-gun, Gunma 370-0503, Japan; matsuura.tomomi.g2@daiichisankyo.co.jp (T.M.); aki.yuichi.ha@daiichisankyo.co.jp (Y.A.); katayama.yuta.nt@daiichisankyo.co.jp (Y.K.); sawada.taihei.zr@daiichisankyo.co.jp (T.S.); kakihara.hirofumi.d8@daiichisankyo.co.jp (H.K.); nonaka.koichi.k2@daiichisankyo.co.jp (K.N.)

**Keywords:** Mobius^®^ CellReady 3 L, Euler–Lagrange, two-way coupling, mixing time, oxygen mass transfer coefficient, hydrodynamic stress

## Abstract

Two-way Euler-Lagrange simulations are performed to characterize the hydrodynamics in the single-use bioreactor Mobius^®^ CellReady 3 L. The hydrodynamics in stirred tank bioreactors are frequently modeled with the Euler–Euler approach, which cannot capture the trajectories of single bubbles. The present study employs the two-way coupled Euler–Lagrange approach, which accounts for the individual bubble trajectories through Langrangian equations and considers their impact on the Eulerian liquid phase equations. Hydrodynamic process characteristics that are relevant for cell cultivation including the oxygen mass transfer coefficient, the mixing time, and the hydrodynamic stress are evaluated for different working volumes, sparger types, impeller speeds, and sparging rates. A microporous sparger and an open pipe sparger are considered where bubbles of different sizes are generated, which has a pronounced impact on the bubble dispersion and the volumetric oxygen mass transfer coefficient. It is found that only the microporous sparger provides sufficiently high oxygen transfer to support typical suspended mammalian cell lines. The simulated mixing time and the volumetric oxygen mass transfer coefficient are successfully validated with experimental results. Due to the small reactor size, mixing times are below 25 s across all tested conditions. For the highest sparging rate of 100 mL min${}^{-1}$, the mixing time is found to be two seconds shorter than for a sparging rate of 50 mL min${}^{-1}$, which again, is 0.1 s longer than for a sparging rate of 10 mL min${}^{-1}$ at the same impeller speed of 100 rpm and the working volume of 1.7 L. The hydrodynamic stress in this bioreactor is found to be below critical levels for all investigated impeller speeds of up to 150 rpm, where the maximum levels are found in the region where the bubbles pass behind the impeller blades.

## 1. Introduction

A typical application of sparged stirred tank bioreactors is the production of antibodies via the cultivation of mammalian cells. Recently, single-use cultivation vessels have become more and more popular compared to multi-use bioreactors because they avoid cleaning and sterilization steps between the cultivation runs. Cell growth and productivity are affected by the flow characteristics in the bioreactor, including concentration gradients, mixing time, oxygen availability, and shear stress [1,2,3,4].

Typically, physically resolved information on these parameters is not available from experiments so that empirical correlations are derived and used to determine the volumetric oxygen mass transfer coefficient, the shear stress, or the mixing time [1,5,6,7,8,9,10,11]. However, these correlations are restricted to the bioreactor under consideration and are not generally valid. In this situation, computational fluid dynamics (CFD) simulations are recommended to obtain both time and spatially resolved information on the above mentioned process characteristics [12,13,14,15,16] also in view of scale-up to larger bioreactors [17,18,19,20].

The flow in a stirred tank bioreactor can be described as a bubbly flow, agitated by an impeller. The most commonly used approach to model this type of flow is the Euler–Euler (EE) approach [15,16,17,21,22], where two sets of continuous phases are considered that penetrate each other. The advantage of this approach is the possibility of the consideration of the head space of the reactor and the liquid surface. Another approach is the Euler–Lagrange (EL) approach, where the liquid phase is treated as continuous and the gas bubbles are tracked through Lagrangian equations. This approach is more commonly found in the simulation of bubble columns [23,24,25] and has rarely been used to model stirred tank bioreactors [26,27,28].

Weber and Bart [29] performed a direct comparison of EE and EL simulations of a bubbly flow in a two-dimensional bubble column and they found reasonable agreement between both simulation approaches and experimental data. Werner et al. [13] recommend to use EE simulations when the dispersed phase volume fraction is larger than 10%, which is hardly reached in bioreactors used for the purpose of mammalian cell cultivation. The present study considers the two-way coupled Euler–Lagrange approach for the single-use Mobius^®^ CellReady 3 L (CR3) bioreactor, where the volume fractions of the gas phase are below 10% in 99.8% of the computational domain.

The Mobius^®^ CellReady 3 L is a single-use, lab-scale, stirred tank bioreactor with a rigid transparent plastic vessel and is equipped with a marine-blade impeller and a microporous sparger as well as an open pipe sparger. Lab-scale bioreactors are important during the process development and for the cell expansion required to inoculate larger bioreactors. Odeleye et al. [10] experimentally investigated the liquid flow in the CR3 with particle image velocimetry (PIV) using silvered hollow glass spheres with a diameter of 10 μm that are carried along with the liquid motion. They cultivated Chinese Hamster Ovary (CHO) cells at three different combinations of impeller speed and working volume, i.e., 80 rpm and 2.4 L, 200 rpm and 2.4 L as well as 350 rpm and 1 L, corresponding to low, medium, and high liquid turbulence, and they found an increased lag-phase and slightly reduced cell size for the high-turbulence condition. Kaiser et al. [21] analyzed the mixing time in the CR3 through both simulations and experiments, where only the liquid phase was considered. They also measured the volumetric oxygen mass transfer coefficient, $k_{L}a$ and compared it to EE simulation results for sparging with the microporous sparger in a sodium sulfate solution. Kaiser et al. [21] concluded that the single-use bioreactor CR3 provides $k_{L}a$ values comparable to similar multi-use vessels and that the low volumetric power input suggests low mechanical stress and therefore a high suitability of the CR3 for the cultivation of shear-sensitive cells. Wutz et al. [26] evaluated the $k_{L}a$ with the two-way coupled EL approach and experiments for a 2.3 L baffled bioreactor with three impellers with four vertical blades and a ring sparger, which makes it different from the presently studied CR3 bioreactor.

The present study addresses the hydrodynamics in the Mobius^®^ CellReady 3 L bioreactor with the two-way coupled Euler–Lagrange approach; thus, it is the first to investigate the trajectories of the individual bubbles in the CR3. The mixing time, the $k_{L}a$, and the hydrodynamic stress are evaluated in the two-phase simulations for different working volumes, impeller speeds, sparging rates, and for the microporous and the open pipe spargers. The two-way coupling of the phases includes the effect of sparging on the mixing time and the hydrodynamic stress. Experiments to evaluate the $k_{L}a$ and the mixing time are also performed and the results are compared with these of the simulations. Moreover, the effect of the different sparger types on the $k_{L}a$ is investigated. The risk of cell damage is evaluated in terms of the Kolmogorov length scale and the hydrodynamic stress, and the consequence on the selection of process conditions is discussed from the perspective of cell culture process optimization.

The following sections provide details of the bioreactor, its operating conditions, and the experiments as well as the modeling approach. Moreover, the numerical and experimental results are presented and discussed.

## 2. Configuration, Operating Conditions, and Experimental Methods

This section provides details of the configuration of the single-use Mobius^®^ CellReady 3 L bioreactor, its operating conditions, and the experimental methods used in the present study.

### 2.1. Configuration and Operating Conditions

**Figure 1:**
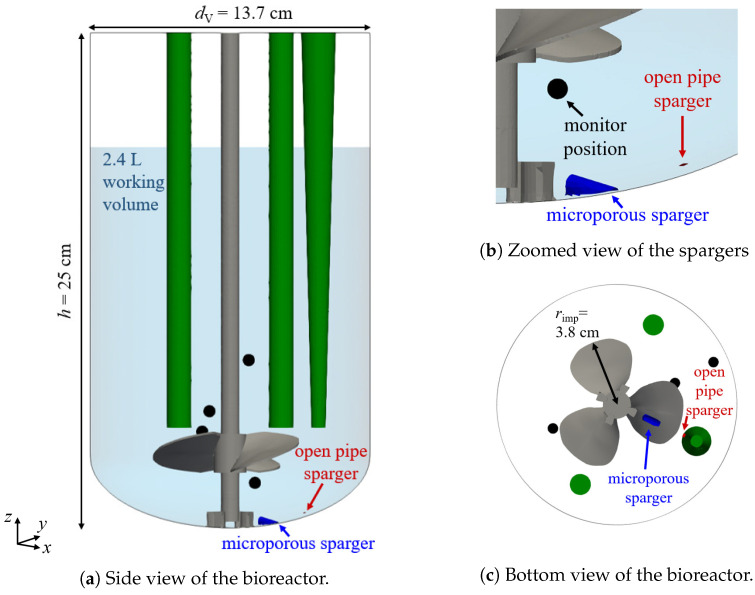
Configuration of the Mobius^®^ CellReady 3 L. The sensors, the microporous sparger, and the open pipe sparger are shown in green, blue, and red color, respectively. The four monitor positions for the mixing time are marked with black bullets.

The Mobius^®^ CellReady 3 L (Merck KGaA, Darmstadt, Germany) is a single-use stirred tank bioreactor with a transparent vessel. Figure 1a displays the configuration and the dimensions. The vessel diameter $d_{V}$ is 13.7 cm and the total height *h* is 25 cm. The view is rotated to show the positions of the three sensors colored in green. The conical sensor is used for the temperature measurement. The pH and the dissolved oxygen tension are determined at the bottom of the left and right cylindrical sensors in Figure 1a, respectively. The reactor has two spargers positioned at the bottom of the vessel highlighted in Figure 1b: an open pipe sparger located at the bottom of the vessel and a microporous sparger consisting of a sintered material with pore sizes in the range of 15 μm to 30 μm. There are four monitor positions used in the simulations to evaluate the mixing time. They are marked with black bullets, all four are visible in Figure 1a, one in Figure 1b, and in Figure 1c, one is hidden by the impeller. The bioreactor is equipped with a marine-blade impeller with three blades, and the impeller diameter $d_{\mathrm{imp}}$ is 7.6 cm, as shown in Figure 1c.

The hydrodynamic characteristics of the bioreactor are dominated by the agitation of the liquid through the clockwise motion of the impeller and the motion of the dispersed bubbles generated by the spargers. Accordingly, the cell free continuous liquid and the disperse bubbles are considered. The effect of modifying the working volume, the sparging rate, the sparger type, and the impeller speed on the mixing time, the oxygen mass transfer, and on the hydrodynamic stress is investigated. Experiments and simulations are performed for the eight different operating conditions given in Table 1. The base case with the intermediate levels is condition #4 from which each parameter is increased or decreased, leading to seven different conditions, and the sparger type is changed in condition #8.

### 2.2. Experimental Methods

The experiments in the bioreactor include the mixing time and the volumetric oxygen mass transfer coefficient, which are used for comparison with the simulations. All measurements are taken in triplicate. In the results’ section, the mean value and the standard deviation evaluated from the three experimental verifications of the measured values are presented.

The mixing time is determined with the iodine de-colorization method [11]. The bioreactor is filled with tap water, which is colored by addition of a combination of 4 mL per 1 L of starch solution (10 g L${}^{-1}$ soluble starch and 2 g L${}^{-1}$ benzoic acid) and 1 mL per 1 L iodine solution (400 g L${}^{-1}$ potassium iodine and 127 g L${}^{-1}$ iodine). The mixing time is taken with a stopwatch as the time from the addition of 0.1 mL per 1 L thiosulfate (166 g L${}^{-1}$ sodium thiosulfate) solution from the top until the liquid is completely clear.

The volumetric oxygen mass transfer coefficient $k_{L}a$ is determined with the dynamic method [7,11]. After oxygen is stripped from the liquid, the volumetric oxygen mass transfer coefficient $k_{L}a$ is evaluated from the variation of the dissolved oxygen tension with time during sparging with oxygen according to [7]

(1) $$\ln\left(\frac{{\mathrm{DO}}^{*}-{\mathrm{DO}}_{t}}{{\mathrm{DO}}^{*}-{\mathrm{DO}}_{0}}\right)=-k_{L}a\left(t-t_{0}\right),$$

where ${\mathrm{DO}}_{0}$, ${\mathrm{DO}}_{t}$, and ${\mathrm{DO}}^{*}$ are the dissolved oxygen tension at the initial time $t_{0}$, at time *t*, and at saturation denoted by ${}^{*}$, respectively. The measurements are performed in a model medium (6 g L${}^{-1}$ NaCl, 1 g L${}^{-1}$ Poloxamer 188, and 0.05 g L${}^{-1}$ active silicone dissolved in purified water) at 37 ${}^{\circ }$C with a head sweep airflow of 100 mL min${}^{-1}$. This procedure is similar to the setup described in previous studies [30,31] concerning the XDR 10 and the XDR 200 bioreactors.

## 3. Mathematical Model

The cell culture medium is treated as a continuous liquid phase and the dispersed phase consists of the gas bubbles. The liquid density, $\rho_{l}$ = 1010.8 kg m${}^{-3}$, and the kinematic viscosity, $\nu_{l}\;=1.114\times 10^{-6}$ m${}^{2}$ s${}^{-1}$, are evaluated from the experiment and are assumed to be constant. The impeller Reynolds numbers, Re = $nd_{S}^{2}/\nu_{l}$, where *n* denotes the impeller speed, are 4344 at 50 rpm, 8687 at 100 rpm, and 13,031 at 150 rpm and lie within the turbulent flow regime. The oxygen bubbles generated at the submerged spargers are considered to remain spherically symmetric throughout the flow.

The continuous liquid phase is described by Eulerian equations and the bubbles are dispersed and treated in a Lagrangian way. Two-way coupling of the phases is achieved through source terms in the Eulerian equations that account for the interaction between the two phases.

The k−ε model has been applied quite frequently to account for the turbulent flow in stirred tank reactors [21,22,26,32,33], and it is also used in the present study.

### 3.1. Continuous Phase

The unsteady Reynolds-averaged Navier–Stokes (URANS) conservation equations of mass and momentum of an incompressible continuous fluid with volume fraction $\alpha_{l}$, where the turbulence is described by the k−ε model, yield

(2) $$\frac{\partial \alpha_{l}}{\partial t}+\nabla \cdot \left(\alpha_{l}u\right)=0$$

(3) $$\frac{\partial \left(\alpha_{l}u\right)}{\partial t}+\nabla \cdot \left(\alpha_{l}u⊗u\right)=-\nabla \left(\frac{p}{\rho_{l}}\right)+\nabla \cdot \left(\alpha_{l}R_{\mathrm{eff}}\right)+\alpha_{l}g+S$$

where u and $\rho_{l}$ are the liquid velocity and density, respectively, *p* is the static pressure, $R_{\mathrm{eff}}$ denotes the effective stress tensor, g is the gravitational acceleration, and S denotes the momentum exchange with the dispersed phase.

The effective stress $R_{\mathrm{eff}}$ is composed of the viscous and the Reynolds stresses, where the effective dynamic viscosity, $\mu_{l,\mathrm{eff}}=\mu_{l}+\mu_{l,t}$, is the sum of the dynamic fluid viscosity and the turbulence dynamic viscosity. The latter is evaluated from the turbulence kinetic energy *k* and its dissipation rate ε as $\mu_{l,t}=\rho_{l}C_{\mu }k^{2}/\varepsilon$, where $C_{\mu }=0.09$ is a model constant. The turbulence kinetic energy $k=\frac{1}{2}\sum_{i=1}^{3}\bar{{u_{i}^{\prime }}^{2}}$ and its dissipation rate $\varepsilon =\nu_{l}\bar{\frac{\partial u_{i}^{\prime }}{\partial x_{j}}\frac{\partial u_{i}^{\prime }}{\partial x_{j}}}$, are evaluated from separate transport equations [34,35]; $u_{i}^{\prime }$ and $u_{j}^{\prime }$ denote fluctuating velocity components in physical *i* and *j* directions, respectively, and $x_{i}$ and $x_{j}$ are the corresponding physical coordinates, i,j=1,2,3.

The transport equations of the turbulence kinetic energy *k* and its dissipation rate ε are described by

(4) $$\begin{array}{c} \frac{\partial \left(\alpha_{l}\rho_{l}k\right)}{\partial t}+\nabla \left(\alpha_{l}\rho_{l}uk\right)=\nabla \left(\alpha_{l}\left[\mu_{l}+\frac{\mu_{t}}{\sigma_{k}}\right]\nabla k\right)+\alpha_{l}G_{k}-\alpha_{l}\rho \varepsilon \end{array}$$

(5) $$\begin{array}{c} \frac{\partial \left(\alpha_{l}\rho_{l}\varepsilon \right)}{\partial t}+\nabla \left(\alpha_{l}\rho_{l}u\varepsilon \right)=\nabla \left(\alpha_{l}\left[\mu_{l}+\frac{\mu_{t}}{\sigma_{\varepsilon }}\right]\nabla \varepsilon \right)+C_{1\varepsilon }\alpha_{l}\frac{\varepsilon }{k}G_{k}-C_{2\varepsilon }\alpha_{l}\rho \frac{\varepsilon^{2}}{k} \end{array}$$

where the model coefficients $\sigma_{k},\sigma_{\varepsilon },C_{1\varepsilon }$ and $C_{2\varepsilon }$ are 1.0, 1.3, 1.44, and 1.92, respectively, and $G_{k}$ is the turbulence production term

(6) $$G_{k}=R_{t}\cdot \nabla u=\left(\mu_{l,t}\left[\nabla u+\nabla u^{T}-\frac{2}{3}I\nabla u\right]-\frac{2}{3}\rho_{l}Ik\right)\nabla u,$$

where I is the identity matrix.

### 3.2. Bubble Phase

The bubbles are treated in a Lagrangian way, i.e., the bubble trajectories are modeled and matched to the Eulerian grid of the liquid phase through the bubble velocity $u_{b}$ as

(7) $$\frac{dx_{b}}{dt}=u_{b},$$

where $x_{b}$ is the position of the bubble.

The velocity of any bubble, b, is evaluated from Newton’s second law of motion

(8) $$\begin{array}{ccc} m_{b}\frac{\partial u_{b}}{\partial t} & = & \underset{\mathrm{drag}\;\mathrm{force},\;F_{D,g}}{\underbrace{\frac{3}{4}C_{D}V_{b}\frac{\rho_{l}}{d_{b}}\left(u-u_{b}\right)\left|u-u_{b}\right|}}\\ & + & \underset{\mathrm{virtual}\;\left(\mathrm{or}\;\mathrm{added}\right)\;\mathrm{mass},\;F_{\mathrm{VM},g}}{\underbrace{C_{\mathrm{VM}}V_{b}\rho_{l}\left(\frac{Du}{Dt}-\frac{Du_{b}}{Dt}\right)}}\\ & + & \underset{\mathrm{lift}\;\mathrm{force},\;F_{L,g}}{\underbrace{C_{L}V_{b}\rho_{l}\left(u-u_{b}\right)\times \left(\nabla \times u\right)}}\\ & + & \underset{\mathrm{gravitational}\;\mathrm{force}}{\underbrace{V_{b}g\;\left(\rho_{g}-\rho_{l}\right)}}+\underset{\mathrm{pressure}\;\mathrm{gradient}\;\mathrm{force}}{\underbrace{V_{b}\rho_{l}\frac{Du}{Dt}}} \end{array}$$

with the bubble mass $m_{b}=V_{b}\rho_{g}$, where $V_{b}$ denotes the bubble volume. $\frac{D}{Dt}$ is the material or substantial derivative, $\rho_{g}$ is the gas density and the right-hand side of the equation represents the forces acting on the bubbles. The drag coefficient $C_{D}$ is modeled following the Schiller and Naumann [36] equation.

The virtual mass coefficient, $C_{\mathrm{VM}}$ is 0.5 [21,26,37], and the lift coefficient $C_{L}$ is calculated according to the correlation of Tomiyama et al. [38], depending on the bubble Eötvös number $\left(\mathrm{Eo}=\Delta \rho |g|d_{b}^{2}/\sigma_{l}\right)$.

(9) CL=min{0.28tanh0.121Re,f}ifEo≤4fif4<Eo≤10−0.27ifEo>10

where $f=0.00105\;{\mathrm{Eo}}^{3}-0.0159\;{\mathrm{Eo}}^{2}-0.0204\;\mathrm{Eo}+0.474$ [38].

The Basset force, also referred to as history force, which is a time integral due to the past acceleration, is neglected because of its high computational demand [39]. Due to the low density of a bubble and the consequently low moment of inertia, the bubble spin is considered to be equal to the liquid spin and thus, the Magnus force is considered to be negligible. Moreover, it is assumed that there is no variation in surface tension across the bubble surface as they rise from the bottom to the top of the reactor and therefore, the Marangoni effect is also neglected. Wall lubrication, which represents lift effects close to the wall and results in bubbles being pushed away from the wall, is not considered.

Different correlations exist to predict the bubble size based on their formation at the sparger. Jamialahmadi et al. [40] reviewed multiple correlations and proposed one based on dimensionless numbers

(10) $$d_{b}=d_{O}{\left[\frac{5}{{\mathrm{Bo}}_{O}^{1.08}}+\frac{9.261\;{\mathrm{Fr}}^{0.36}}{{\mathrm{Ga}}^{0.39}}+2.147\;{\mathrm{Fr}}^{0.51}\right]}^{1/3},$$

where $d_{b}$ and $d_{O}$ denote the bubble and orifice diameter, respectively. For the microporous sparger, $d_{O}$ is set to the median of the supplier-specified pore size range of 15 to 30 μm. ${\mathrm{Bo}}_{O}=\rho_{l}\left|g\right|d_{O}^{2}/\sigma_{l}$ is the Bond number, Bo, in terms of $d_{O}$, and $\sigma_{l}$ is the surface tension, which is evaluated from experiment as 0.048 N m${}^{-1}$. The Froude number Fr = $|u_{O}{|}^{2}/(d_{O}\left|g|\right)$ is determined from the orifice velocity $u_{O}$ calculated as the sparging rate divided by the total sparger area. Ga = $\rho_{l}^{2}d_{O}^{3}\left|g\right|/\mu_{l}^{2}$ is the Galileo number in terms of $d_{O}$. For bubbles generated by the microporous sparger, the bubble diameter according to Equation (10) is 1 mm and is assumed not to change with the sparging rates considered in the present study, since the change in $u_{O}$ is small. For the open pipe sparger, the bubble diameter is 4 mm for a sparging rate of 50 mL min${}^{-1}$.

To fully capture the actual bubble size distribution as well as breakup and coalescence of bubbles, a population balance modeling approach would be required, which would significantly increase both the model complexity and the computational time [41]. Since the temperature is considered constant and the Mach number is low, no changes in the gas density are expected. Additionally, the change in hydrostatic pressure from the bottom of the reactor up to the liquid surface results in slight changes in the bubble size and the gas density, which is neglected due to the small liquid height of up to 18 cm. For the maximum working volume, the increase in the bubble volume is evaluated to be less than 1.8%.

The number of bubbles inside the domain, $n_{b}$, is initially zero and changes with time according to

(11) $$\frac{dn_{b}}{dt}=r_{\mathrm{gen}}-r_{\mathrm{rem}}.$$

The bubbles are generated at the sparger and tracked until they reach the top of the computational domain, where they exit. The bubble generation rate, $r_{\mathrm{gen}}=Q/V_{b}$ depends on the sparging rate *Q*, see Table 1, and the bubble volume $V_{b}$. If the microporous sparger is used, a bubble diameter of 1 mm is used, which results in a bubble generation rate of 318 s${}^{-1}$, 1592 s${}^{-1}$, and 3183 s${}^{-1}$ for the sparging rates of 10 mL min${}^{-1}$, 50 mL min${}^{-1}$, and 100 mL min${}^{-1}$, respectively. The open pipe sparger with an initial bubble diameter of 4 mm requires a bubble number rate of 25 s${}^{-1}$ at the intermediate sparging rate of 50 mL min${}^{-1}$.

The bubble removal rate, $r_{\mathrm{rem}}$, depends on the number of bubbles that reach the top boundary. At a quasi-steady condition, the average total number of bubbles $n_{b,\mathrm{avg}}$ in the domain is related to the average bubble residence time $\tau_{b,\mathrm{res}}$ and the bubble generation rate $r_{\mathrm{gen}}$ through

(12) $$n_{b,\mathrm{avg}}=\tau_{b,\mathrm{res}}r_{\mathrm{gen}}.$$

In the present study, a two-way coupled approach is applied, where the momentum and volumetric coupling of the bubbles with the liquid phase are considered. The volume fraction of the continuous liquid phase is $\alpha_{l}=1-\alpha_{g}$, where $\alpha_{g}=\sum_{k=1}^{n_{b}}V_{b,k}/V_{\mathrm{CV}}$, denotes the volume fraction covered by the dispersed bubbles in a Eulerian control volume. Here, $n_{b}$ is the number of bubbles inside a control volume, cf. Equation (11), $V_{b,k}$ is the volume of bubble *k*, and $V_{\mathrm{CV}}$ is the control volume.

The source term, S in the momentum equation of the continuous phase, Equation (3), yields

(13) $$\begin{array}{c} S=-\frac{\alpha_{g}}{V_{b}}\left(F_{D,g}+F_{\mathrm{VM},g}+F_{L,g}\right), \end{array}$$

where $F_{D,g}$, $F_{\mathrm{VM},g}$, and $F_{L,g}$ are the drag force, the virtual mass force, and the lift force acting on the bubbles.

### 3.3. Evaluation of the Mixing Time and the Oxygen Mass Transfer Coefficient

For comparison of the numerical simulations with the experimental data, the mixing time and the oxygen mass transfer coefficient are available. Their mathematical evaluation is described in the following.

#### 3.3.1. Mixing Time

The mixing time is calculated from the transport equation of a passive tracer in the liquid with a normalized concentration *c* between zero and unity [15]

(14) $$\frac{\partial \left(\alpha_{l}c\right)}{\partial t}+\nabla \left(\alpha_{l}uc\right)=-\nabla \left(\alpha_{l}D_{\mathrm{eff}}\nabla c\right),$$

where $D_{\mathrm{eff}}=D_{m}+\frac{\nu_{l,t}}{{\mathrm{Sc}}_{t}}$ is the effective liquid diffusivity. The turbulent Schmidt number ${\mathrm{Sc}}_{t}$ is set to 0.7 [32,39] and $\nu_{l,t}$ is the turbulence kinematic viscosity of the liquid. The change of the tracer concentration over time is monitored at four different positions in the reactor, which are marked in Figure 1. In the simulations, the initial volume of the tracer, which is added from the top in the experiments, is defined as a spherical region located directly below the liquid surface with a diameter of 3 cm corresponding to a volume of 14 mL for which c=1. The mixing time is determined as the time at which the concentration at all four positions is within ±5% of the constant final value at steady conditions.

#### 3.3.2. Volumetric Oxygen Mass Transfer Coefficient

The liquid oxygen mass transfer coefficient $k_{L}$ is calculated following the eddy cell model of Lamont and Scott [42]

(15) $$k_{L}=0.4\;\sqrt[{}]{D_{O_{2}}}{\left(\frac{\varepsilon }{\nu_{l}}\right)}^{0.25}.$$

$D_{O_{2}}$ is the diffusion coefficient of oxygen in water ($3\times 10^{-9}$ m${}^{2}$ s${}^{-1}$ at 37 ${}^{\circ }$C) [43], and $k_{L}$ is determined based on the average turbulence energy dissipation rate. The specific interface area *a* is

(16) $$a=n_{b,\mathrm{avg}}\frac{d_{b}^{2}\pi }{V},$$

where $d_{b}^{2}\pi$ is the surface area of an individual bubble and *V* the working volume. The volumetric oxygen mass transfer coefficient $k_{L}a$ is the product of the $k_{L}$ and *a*.

## 4. Numerical Solution Procedure and Grid

The governing equations are solved using the open-source code OpenFOAM version 7 [44]. Details of the implementation will be presented before the numerical grid is displayed and discussed.

### 4.1. Numerical Solution Procedure

The two-way coupled Euler-Lagrange simulations are performed using the DPMFoam solver of the open-source software OpenFOAM [44]. For initializing the simulations, steady state simulation results for the single phase liquid flow are used. The steady state simulations are performed with the simpleFOAM solver using the multiple reference frame approach to model the impeller motion. For the two-way coupled Euler–Lagrange simulations, the impeller motion is modeled using the sliding mesh approach [45]. The simulations are run using the PIMPLE algorithm until steady conditions are reached. The Courant number is assured to remain below 0.2.

**Figure 2:**
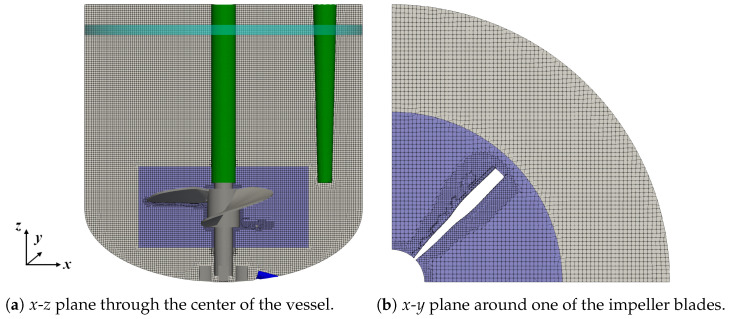
Computational grid for the liquid phase for the working volume of 1.7 L.

The volumetric coupling includes the displacement of liquid by the disperse bubbles. Therefore, the addition and removal of bubbles must be balanced by the in- and outflow of the continuous phase. In the experiment, this results in a small increase in the liquid height, which is too small to be detected. In the simulations, allowing for in- and outflow across the top boundary has a severe impact on the liquid flow, since the liquid height does no longer correspond to the selected working volume. Thus, the top boundary has to be treated with a slip boundary condition to achieve a representation of the liquid surface. A procedure similar to the strategy applied by Masterov et al. [24] in an Euler–Lagrange simulation of a bubble column with a square cross-section is followed to consider the displacement of the liquid through bubbles: An artificial boundary is created by selecting a small section of the vertical reactor wall close to the liquid surface to allow for a pressure-dependent in- and outflow of the liquid. This artificial boundary forms a ring with a height of 0.5 cm along the circumference of the vertical vessel wall, which is indicated by the teal stripe in Figure 2. Another method using an artificial boundary with a reduced area consisting of two stripes of 2 cm width on opposing sides of the reactor wall resulted in a pressure difference between that boundary and the vessel wall and therefore, this method was not investigated further.

### 4.2. Computational Grid

The Eulerian grid for the liquid phase ranges up to the liquid surface, accordingly, and the grid height is adapted to match the working volume. The numerical grid with 1.9 × 10${}^{6}$ cells for the intermediate working volume of 1.7 L is shown in Figure 2a. All three sensors shown in Figure 1 are considered, but for the view point used to present the *x*-*z* plane, the two cylindrical sensors overlap with the central stirrer shaft. The corresponding grids for a volume of 1.0 L and 2.4 L consist of 1.1 × 10${}^{6}$ and 2.6 × 10${}^{6}$ grid cells, respectively. The grids are created with the meshing tool snappyHexMesh available in OpenFOAM [44]. The majority of the grid cells are hexahedral with a grid spacing of 0.2 mm in *x*, *y*, and *z* directions. In close proximity to the impeller, the grid spacing is reduced to 0.1 mm and polyhedral cells are used at the boundaries and to achieve the transition of the grid spacing from 0.2 mm to 0.1 mm. The grid in *x* and *y* directions around one of the impeller blades to the vessel walls is shown in Figure 2b.

Grid independence has been tested with single liquid phase steady-state simulations, and the presented grid is found to be sufficiently refined since it shows less than 2% difference in the average velocity magnitude, the turbulence kinetic energy and its dissipation rate compared to the finest grid with 3.3 × 10${}^{6}$ cells for the 1.7 L working volume.

## 5. Results and Discussion

The first part concerns the flow field, the bubble trajectories, and the gas hold-up. Then, the simulated volumetric oxygen mass transfer coefficient and the mixing time are compared with the experimental results. Finally, the risk of cell damage for the tested operating conditions is investigated.

### 5.1. Flow Characteristics and Gas Hold-Up

**Figure 3:**
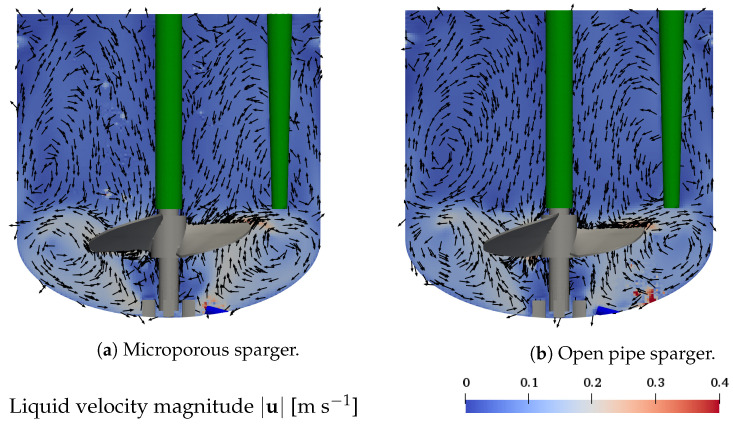
Liquid flow field in the *x*-*z* plane through the center of the vessel. Arrows show the velocity in *x*-*z*-direction. Conditions: *V* = 1.7 L, *n* = 100 rpm, and *Q* = 50 mL min${}^{-1}$. (**a**) Microporous sparger (condition #4) and (**b**) the open pipe sparger (condition #8).

First, the effect of using different spargers on the liquid flow field is studied. Figure 3a,b display the magnitude of the liquid flow velocity in the *x*-*z* plane plane through the center of the vessel as well as the velocity vector in the same plane for the microporous sparger (base case, condition #4) and the open pipe sparger (condition #8), respectively. The sparging rate is 50 mL min${}^{-1}$, the working volume is 1.7 L, and an impeller speed of 100 rpm is considered, as shown in Table 1.

The clockwise rotational motion of the marine-blade impeller results in a dominant clockwise rotational motion of the liquid. The axial and radial flow structures are characterized by two recirculation zones. The liquid moves from the impeller towards the vessel wall, where it is redirected in up- and downward directions. The downward moving liquid again is redirected at the curved bottom towards the center, resulting in a recirculation zone. The upward moving liquid flows along the vessel wall towards the liquid surface, where it is redirected to the vessel center, from where it flows downwards along the centered impeller rod, forming a second, more extended recirculation zone of lower velocity. The liquid velocity in the lower recirculation zone is in the range of 0.1 m s${}^{-1}$ to 0.3 m s${}^{-1}$, whereas in the upper recirculation zone, it is below 0.1 m s${}^{-1}$. The coupling with the disperse bubbles leads to localized fluctuations of the liquid velocity above the sparger and in the regions where bubbles are rising. The flow structures resulting with the use of the microporous sparger and the open pipe sparger are very similar, indicating that the selection of the sparger has little impact on the liquid flow field. The in- and outflow boundary described in Section 4.2 also results in localized velocity fluctuations in the direct proximity of the vessel wall, indicated by the slightly higher velocity in the top part of Figure 3a,b. However, these localized fluctuations do not disrupt the general flow pattern of the two recirculation zones.

The same general liquid flow structure as presented in Figure 3 is found for all other test conditions given in Table 1. A higher impeller speed causes an increase in the liquid velocity magnitude. Changes in the working volume, affecting the height of the computational domain, lead to differences in the height of the upper recirculation zone for which the liquid velocity decreases with increasing distance to the impeller. The size of the lower recirculation zone remains the same across the different conditions, which is associated with the same position of the impeller for the different working volumes under consideration. The described liquid flow structure conforms well to experimental results by Odeleye et al. [10] and simulations by Kaiser et al. [15], who studied the liquid flow field without sparging in the Mobius^®^ CellReady 3 L. The high similarity between these studies and the liquid flow for the two different spargers obtained in the present studies emphasizes that the bubbles only have localized effects on the liquid flow, while the overall liquid flow structure is dominated by the impeller motion.

**Figure 4:**
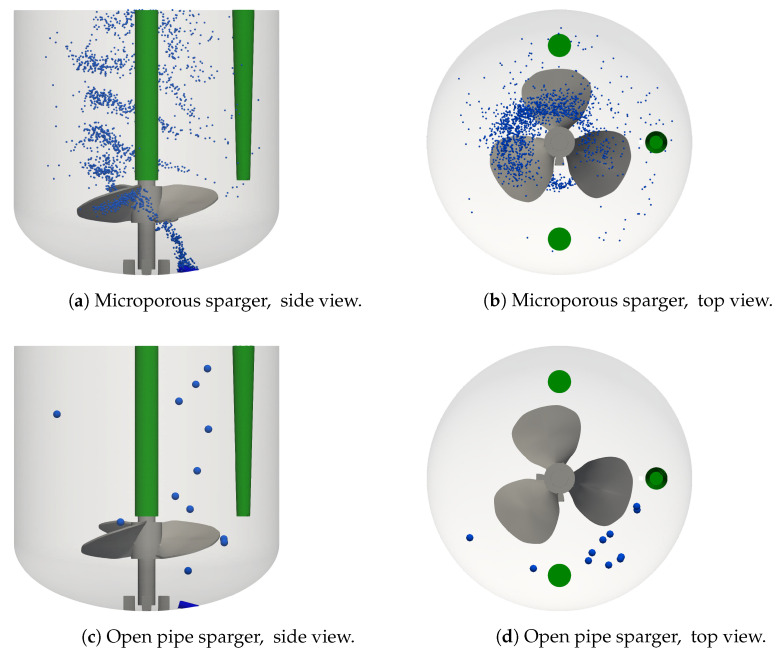
Bubble dispersion for the microporous sparger (**a**,**b**) base case, condition #4, and the open pipe sparger (**c**,**d**) condition #8. *V* = 1.7 L, *n* = 100 rpm, and *Q* = 50 mL min${}^{-1}$.

Representative bubble positions for the base case are shown in Figure 4a,b and those for the corresponding open pipe sparger condition are displayed in Figure 4c,d. Buoyancy and drag forces have a dominant effect on the trajectories of bubbles, resulting in their rise from the sparger to the liquid surface in a clockwise motion following the motion of the liquid flow. The bubbles that are generated by the microporous sparger, see Figure 4a, get caught behind the impeller blades. The bubbles move with the impeller blades in a clockwise direction and rise behind the impeller blade to leave the impeller region on the side opposite to the sparger. Above the impeller, most bubbles rise close to the center of the reactor and at the same time, they move further up in clockwise direction. The horizontal dispersion of the bubbles increases somewhat above the impeller region. This qualitative behavior is in agreement with the study by Kaiser et al. [21], where the Euler–Euler approach is used. However, in the study by Kaiser et al. [21], the multiple reference frame (MRF) approach is used to model the stirring. In the MRF approach, the impeller is not actually moving, so that the clockwise motion of the bubbles following behind the impeller blades cannot directly be observed by Kaiser et al. [21], and the position of the bubble plume above the impeller is shifted in the anti-clockwise direction. The velocity and the rise pathway of the bubbles determine their residence time. The average residence time of the bubbles and the number of bubbles present at steady condition are related through Equation (12). For the base case shown in Figure 4a,b, $n_{b,\mathrm{avg}}$ is 2295 and it ranges from 459 to 4922 across the different operating conditions with the microporous sparger.

The open pipe sparger with a single opening generates larger bubbles and therefore, fewer bubbles are formed than with the microporous sparger at the same sparging rate, as shown in Figure 4a,c. The considerably larger bubbles formed by the open pipe sparger barely interact with the impeller and their rise pathway is closer to the reactor wall due to the position of the sparger, as shown in Figure 4d, which is further away from the impeller compared to the microporous sparger. While the bubbles are also swept along by the clockwise liquid motion, their larger volume and mass results in a more dominant effect of buoyancy. Consequently, the residence time of the bubbles is shorter and their rise path has a steeper inclination compared to the bubbles generated by the microporous sparger. The shorter residence time in combination with the lower bubble generation rate using the open pipe sparger, cf. Equation (12), result in only twelve bubbles residing in the bioreactor at a quasi steady state.

**Figure 5:**
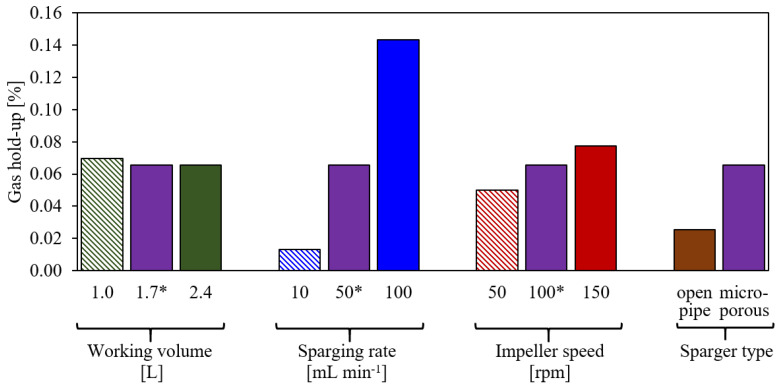
Simulated gas hold-up for the different test conditions given in Table 1. Labels on the abscissa show the modified parameters compared to the base case*.

The gas hold-up is evaluated as the cumulative volume of all bubbles present in the liquid at a steady condition divided by the total volume. Figure 5 summarizes the gas hold-up for the different test conditions, as shown in Table 1. A change in working volume hardly affects the gas hold-up; it decreases somewhat with the increased working volume. The sparging rate shows the strongest effect on the gas hold-up as anticipated, and an increase in impeller speed increases the gas hold-up. The use of the open pipe sparger causes a lower gas hold-up, which is due to the lower residence time of the larger bubbles. The gas hold-up is rather low across all test conditions, i.e., it is below 0.16%, and thus, it cannot be measured through changes in the liquid height. Since the gas hold-up has a strong impact on the $k_{L}a$, a comparison of the simulated and the measured $k_{L}a$ in Section 5.2 will be taken as an indicator of the accuracy of the simulated gas hold-up.

### 5.2. Volumetric Oxygen Mass Transfer Coefficient and Mixing Time

**Figure 6:**
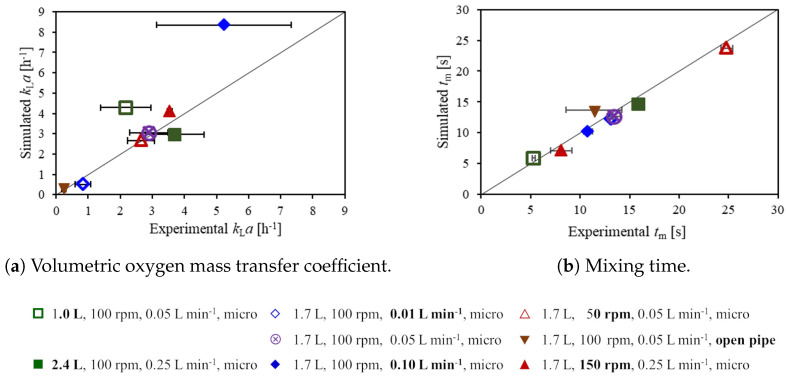
Volumetric oxygen mass transfer coefficient $k_{L}a$ (**a**) and mixing time $t_{m}$ (**b**). Horizontal error bars indicate the standard deviation of the experimental results.

In this subsection, the experimental and computational values of the volumetric oxygen mass transfer and the mixing times are presented, which are shown in Figure 6a. Experiments are preformed in triplicate and the standard deviation of these measurements is reported in the figure. The experimental values of the $k_{L}a$ are between 0.2 ± 0.1 h${}^{-1}$ and 5.2 ± 2.1 h${}^{-1}$, where the lowest $k_{L}a$ across all conditions is found for the open pipe sparger (triangle down) for which the sparging rate is 50 mL min${}^{-1}$. Even for the minimum sparging rate of 10 mL min${}^{-1}$, see Figure 6a (open diamond), the measured $k_{L}a$ is higher than that of the open pipe sparger at 50 mL min${}^{-1}$, despite the lower gas hold-up, as shown in Figure 5. This emphasizes the strong effect of the sparger type on the bubble size and consequently the $k_{L}a$. For a fixed gas flow rate, the bubble size affects the number of bubbles and the specific interface area of $k_{L}a$ in two ways: The bubble residence time increases for smaller bubbles, which, in turn, leads to a higher gas hold-up. Moreover, the $k_{L}a$ is affected by the impact of the bubble size on the specific interface area: Even if the same gas hold-up was achieved with different bubble sizes, the total number of bubbles and the surface area to volume ratio would increase for smaller bubbles. Consequently, the use of the microporous sparger results in a higher $k_{L}a$ compared to the use of the open pipe sparger.

A higher sparging rate not only increases the gas hold-up, as shown in Figure 5, but also the $k_{L}a$ displayed in Figure 6a, where sparging rates of 10 mL min${}^{-1}$, 50 mL min${}^{-1}$ (base case), and 100 mL min${}^{-1}$ are indicated by the open diamond, the crossed circle, and the filled diamond, respectively. This is in agreement with the experimental results. For the maximum sparging rate of 100 mL min${}^{-1}$, the highest deviation between the simulation and the experiment is observed, with simulated and experimental $k_{L}a$ values of 8.4 h${}^{-1}$ and 5.2 ± 2.1 h${}^{-1}$, respectively. The simulation might slightly over-predict the $k_{L}a$ for this condition since the the number of bubbles and the gas hold-up increase with the sparging rate, see Figure 5, and consequently, the probability of coalescence of bubbles increases. The simulations do not account for coalescence, which will increase the average bubble size towards the liquid surface. The agreement between the simulation and the experiment would probably be improved by choosing a slightly larger average bubble size in the simulations, which would reduce the value of the simulated $k_{L}a$.

For a higher impeller speed, a slight increase in gas hold-up (see Figure 5) and in $k_{L}a$ (triangles up and crossed circle, see Figure 6a) is observed. The higher impeller speed also leads to a higher liquid velocity and a higher turbulence energy dissipation rate and thus, the liquid transfer coefficient $k_{L}$ increases. This is observed in both the simulations and the experiments. However, the effect of the impeller speed on the $k_{L}a$ is minor compared to that of the sparging rate and the sparger type. In the experiments, the significance of the increase in $k_{L}a$ is tested with a two-tailed two sample *t*-test and it is too small to be significant for all three possible pairs of impeller speeds with a 95% confidence interval [46].

In contrast to the sparger type, sparging rate, and impeller speed, the effect of the working volume is not so clear. The experimental mean values of $k_{L}a$ slightly increase with the working volume, see Figure 6 (squares and base case); however, the differences are not significant as confirmed by the *t*-test. In the simulations, the gas hold-up is almost the same for the different working volumes, and the $k_{L}$ is slightly higher for a lower working volume due to a higher average turbulence energy dissipation rate, and accordingly, slightly higher $k_{L}a$ values are found for smaller working volumes. Overall, changes in the working volume have a minor impact on the $k_{L}a$.

With regard to the selection of cell culture process conditions, the $k_{L}a$ values observed in the present study are on the lower end of typical cell culture requirements, which are about 1 h${}^{-1}$ to 20 h${}^{-1}$ [1,16,21]. Furthermore, sparging with the open pipe sparger is insufficient to meet cell culture requirements. Using the microporous sparger, the sparging rate may have to be increased beyond the presently investigated range for cell lines with high cell density or high oxygen demand.

A comparison of computational and experimental mixing times $t_{m}$ is presented in Figure 6b. Experimental values are between 5.3 ± 0.2 s and 24.8 ± 0.6 s. The impeller speed and the working volume have a strong impact on the mixing time, as expected. Both an increase in the impeller speed, see Figure 6b (triangles up and crossed circle), and a reduction of the working volume (squares and crossed circle) significantly reduce the mixing time due to both a higher liquid velocity and increased turbulence at a higher impeller speed as well as a smaller size of the upper recirculation zone, and a higher velocity of the upper recirculation zone for a reduced working volume. The effect of the sparging rate on mixing time is smaller than these of the impeller speed and the working volume. For a sparging rate of 100 mL min${}^{-1}$, see Figure 6b (filled diamond), a significant reduction in the mixing time compared to that of the base case, see Figure 6b (crossed circle), is observed in the experiments. The shorter mixing time is most likely caused by the additional liquid motion and turbulence induced by the bubbles in the upper part of the bioreactor. For condition #5, the average turbulence kinetic energy, the turbulence energy dissipation rate, and the turbulence kinematic viscosity in the upper part of the liquid are increased compared to the base case condition #4 by factors of 2.9, 3.4, and, 1.9, respectively. A reduced mixing time with increasing gas flow rate for slow mixing at low impeller speeds has also been reported by Montante and Paglianti [47]. In the present experiments, a significant effect of the sparger type cannot be identified due to the high standard deviation of the measurements for the open pipe sparger, see Figure 6b (triangle down), which is larger than the difference in the mixing times observed with the microporous sparger even for different sparging rates, see Figure 6b (diamonds and crossed circle). In the simulations, the mixing time for the open pipe sparger is very similar to that for the microporous sparger at the same sparging rate, indicating that the sparger type has only a minor effect on the mixing time. This agrees well with the high similarity of the liquid flow patterns for the two different types of spargers, see Figure 3. Overall, the agreement between simulated and experimental mixing times is quite good with a $R^{2}$ value of 0.96.

Kaiser et al. [15] reported mixing times without sparging for 1.5 L at 100 rpm and 150 rpm and for 2.5 L at 100 rpm. The conditions are similar to conditions #4, #7, and #2 of the present study (1.7 L, 100 rpm and 150 rpm as well as 2.4 L, 100 rpm, see Table 1) for which mixing times with sparging are measured. The mixing times reported by Kaiser et al. [15] of 14.9 ± 0.9 s, 7.3 ± 1.2 s, and 27.0 ± 1.8 s, respectively, agree well to those of 13.4 ± 0.7 s, 8.08 ± 1.1 s, and 24.8 ± 0.6 s, respectively, found in the present study.

Due to the small bioreactor size, mixing times are quite short compared to those found for larger bioreactors [5,48]. However, the impeller speed significantly impacts the mixing time and thus, the value of 50 rpm with an almost three times higher mixing time than that for 150 rpm appears to be less favorable for cell cultivation than the higher impeller speed.

### 5.3. Risk of Cell Damage

Cell damage may be caused by different processes, one of which is the mechanical damage of the cell by the liquid. Different methods to calculate the stresses acting on the cells from CFD results are reported in the literature [1,12,13,14,18,49]. Soos et al. [14] proposed a method to calculate the hydrodynamic stress using the turbulence energy dissipation rate ε as

(17) $$\tau_{l}=\sqrt{\rho_{l}\mu_{l}\varepsilon }=\mu_{l}\tau_{K}^{-1},$$

considering the inverse of the Kolmogorov time scale $\tau_{K}$. In other studies [1,12,13,49], the Kolmogorov length

(18) $$l_{K}={\left(\nu_{l}^{3}/\varepsilon \right)}^{0.25}$$

is directly compared to the cell size, as cells are considered only to be damaged if the Kolmogorov length is equal or smaller than the cell size.

The Kolmogorov length and the hydrodynamic stress are related through

(19) $$\tau_{l}=\frac{\mu_{l}\nu_{l}}{l_{K}^{2}},$$

eliminating the turbulence scalar dissipation rate from Equations (17) and (18).

In order to evaluate thresholds for either condition, experimental values for the hydrodynamic stress and the Kolmogorov length are required. However, there is limited information on these values and none of the studies provides both; therefore, a judgement about the preferred method is not possible.

Neunstoecklin et al. [50] experimentally determined thresholds for the maximum tolerable hydrodynamic stress of 25.2 ± 2.4 Pa and 32.4 ± 4.4 Pa for mouse hybridoma Sp2/0 and CHO cells, respectively. Neunstoecklin et al. [50] give a typical size range for mouse hybridoma Sp2/0 and CHO cells of 11–16 μm and 15–18 μm, and Kaiser et al. [15] and Gelves et al. [49] provide typical values of the size range of mammalian cells of 15–20 μm. Following Equation (19), the value of the hydrodynamic stress of 25.2 Pa relates to a Kolmogorov length scale of 7 μm. However, if the typical cell size of 20 μm is used, the corresponding hydrodynamic stress would be 3.14 Pa. Thus, the use of the Kolmogorov length as criterion for cell damage provides considerably lower values than that for the hydrodynamic stress. Since no experimental evidence exists for the relation between the Kolmogorov length and cell viability, the question arises whether the cell size can directly be related to the critical Kolmogorov length.

**Figure 7:**
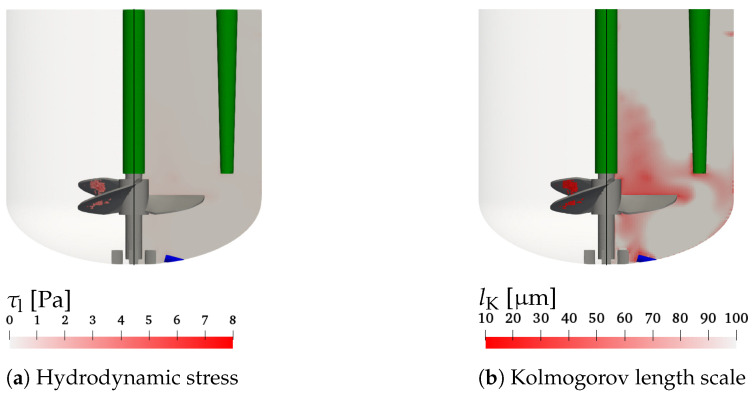
Hydrodynamic stress (**a**) and Kolmogorov length scale (**b**) for *V* = 1.7 L, *n* = 150 rpm, and *Q* = 50 mL min${}^{-1}$ (condition #7). Left parts: control volumes with $\tau_{l}\;>$ 3.14 Pa and $l_{K}<\;20\;\mu$m, respectively. Right parts: values on the *x*-*z* plane through the center of the vessel.

Nevertheless, Figure 7a,b show the hydrodynamic stress and the Kolmogorov length scale, respectively, for condition #7 with the highest impeller speed, which is considered the most critical for cell damage. The left parts of Figure 7a,b visualizes the computational cells with the highest and smallest values of $\tau_{l}$ and $l_{K}$, respectively, which are critical for the cell damage. The right hand sides show the hydrodynamic stress and the Kolmogorov length scale on the *x*-*z* plane through the center of the vessel. It can be observed that the critical regions of the maximum hydrodynamic stress and minimum Kolmogorov length scale are found near the impeller blades. Figure 7a shows that the hydrodynamic stress is well below the critical levels reported by Neunstoecklin et al. [50]. This is also in agreement with cell culture experiments by Odeleye et al. [10], where no adverse effect on the growth of CHO cells is observed for an impeller speed of 200 rpm. However, when considering the Kolmogorov length scale displayed in Figure 7b, a small number of computational cells in the region where bubbles rise behind the impeller blades with Kolmogorov length scales between 10 μm and 20 μm is found, which is comparable to the size of mammalian cells of 15–20 μm [15,49]. It would be interesting to see measurements of the critical hydrodynamic stress and the Kolmogorov length scale for the same cell line to investigate and evaluate the discussed thresholds using both methods.

Another method to evaluate the risk of cell damage was recently suggested by Li et al. [18], who used the strain rate in different zones of the bioreactor. They distinguish the impeller and the tank zone and identify suitable ranges of about 6 s${}^{-1}$ to 10 s${}^{-1}$ for the strain rate in the impeller zone and 2.2 s${}^{-1}$ to 3.6 s${}^{-1}$ in the tank zone for the cultivation of *Spodoptera frugiperda* Sf9 insect cells in 7.5 L to 1000 L bioreactors. In the present study, the impeller zone is defined as a cylindrical volume around the impeller with a height of 0.33 $d_{\mathrm{imp}}$ and a diameter of 1.2 $d_{\mathrm{imp}}$. The cylinder height is the same as reported by Li et al. [18] for pitched blade and propeller type impellers, while the diameter corresponds to that given by Li et al. [18] for ‘Elephant Ear’ impellers, to exclude the sensors from the impeller zone. The tank zone is the computational domain excluding the impeller zone. The average strain rate is evaluated separately in these zones.

**Figure 8:**
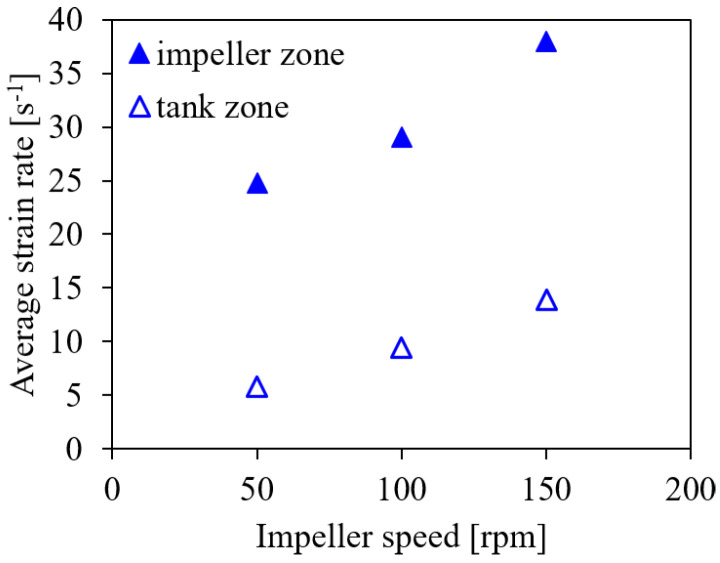
Average strain rate in the impeller and the tank zones for *V* = 1.7 L, *Q* = 50 mL min${}^{-1}$ and the microporous sparger for different impeller speeds of 50 rpm, 100 rpm, and 150 rpm (conditions #6, #4, and #7).

Figure 8 shows the average strain rate for the impeller and the tank zones for the different impeller speeds. The average values of the strain rates in both zones are higher than the limits suggested by Li et al. [18]. However, based on the hydrodynamic stress limits provided by Neunstoecklin et al. [50] for CHO and Sp2/0 cells and the cultivation results of Odeleye et al. [10] for CHO cells, the investigated range of impeller speeds appears to be suitable for both cell lines. This deviation to the criteria derived by Li et al. [18] suggests that either the critical limits need readjustment for working volumes below 7.5 L and different impeller types or that Sf9 cells are more sensitive than CHO and hybridoma cells.

The study by Li et al. [18] motivates the question in how far the size of the volume in which critical values of hydrodynamic stress are violated and is significant in evaluating the risk of cell damage. Moreover, the time during which cells are exposed to these critical levels may be short, the number of the cells in this region may be small, and the frequency at which they travel through this region may be low. Since the cells are able to recover from sub-lethal conditions, the question arises how the volume fraction associated with the violation of a certain critical conditions may affect the cell cultivation. In this regard, considering distributions of the volume fractions of different stress levels [22] or exposure time profiles [51] appear to be more appropriate than using global minimum or maximum values. Moreover, Li et al. [18] found the maximum strain rate not to be indicative of suitable and adverse cell culture process conditions. Instead, they considered the average strain rate in the impeller zone in combination with that in the tank zone as well as the average strain rate of the entire liquid to identify appropriate operating ranges for scale-up. A restriction to certain areas in the bioreactors where the cells are more likely to be damaged than in others as well as the consideration of the time during which the cells are exposed to the high-risk regions in the vessel might lead to interesting considerations with respect to the risk of cell damage. However, any new criterion for the risk of cell damage that is evaluated from CFD simulations, requires evidence from cell culture experiments to determine cell line specific thresholds.

## 6. Summary and Conclusions

This study presents two-way coupled Euler-Lagrange simulations of the cell-free two-phase flow in the lab-scale single-use stirred tank bioreactor Mobius^®^ CellReady 3 L. This bioreactor has a marine blade impeller and two sparger options, i.e., an open pipe sparger and a microporous sparger.

The Lagrangian treatment of the bubbles resolves the individual bubble trajectories. The volumetric coupling of the disperse bubbles with the incompressible liquid requires the compensation of the generation of bubbles at the sparger and their removal at the liquid surface through an in- and outflow of the liquid. To achieve this, a ring-shaped artificial boundary at the upper part of the vessel wall is added, following the study of Masterov et al. [24] for a square bubble column. The sliding mesh approach is used to model the impeller motion, which allows for a detailed understanding of the bubbles’ motion in the impeller region.

The simulated liquid flow structure with recirculation zones above and below the impeller agrees well with the experimental liquid flow structure from particle image velocimetry reported by Odeleye et al. [10] and single liquid-phase simulations by Kaiser et al. [21]. In the present study, it is found that the bubbles have a localized effect on the liquid velocity and the turbulence.

Experiments have been performed to determine the mixing time and the volumetric oxygen mass transfer coefficient for comparison with the numerical results. In contrast to the study by Kaiser et al. [21], the present simulations evaluate the mixing time for sparged conditions. The effect of bubbles on the liquid motion results in a 2 s shorter mixing time at the maximum sparging rate of 100 mL min${}^{-1}$ compared to the intermediate sparging rate of 50 mL min${}^{-1}$ with good agreement between the simulations and the experiments. This indicates that the effect of sparging on the liquid mixing is well-captured in the present simulations. However, the impact of the sparging rate on the mixing time is not as significant as that of the working volume and the impeller speed. The tremendous effect of the sparger type on the volumetric oxygen mass transfer is caused by the difference in the bubbles’ size generated by microporous and the open pipe sparger. Since the bubble formation at the sparger is not part of the simulations, fixed bubble diameters of 1 mm and 4 mm are used for the microporous and the open pipe sparger, respectively, based on the empirical correlation provided by Jamialahmadi et al. [40]. The volumetric oxygen mass transfer coefficient for the open pipe sparger is about one tenth of that for the microporous sparger in agreement with the experimental data. It might be useful to study polydisperse bubble size distributions in future studies to elaborate more on this topic.

Regarding the selection of operating conditions for the cultivation of suspended mammalian cells, the small working volume in the CR3 allows for fast mixing across all operating conditions. However, the mixing time is faster by more than 10 s when the impeller speed is increased from 50 rpm to 100 rpm, indicating that an impeller speed of 100 rpm is preferable. Moreover, low hydrodynamic stress levels for the tested impeller speeds of 100 and 150 rpm indicate no risk of cell damage based on the evaluation of the hydrodynamic stress. To achieve $k_{L}a$ values that are high enough to support typical cell culture requirements, the microporous sparger is preferable compared to the open pipe sparger. The results of the present study can be used to select the operating conditions according to the requirements for the cultivation of a given cell line. Overall, the Mobius^®^ CellReady 3 L as a lab-scale bioreactor is found to provide fast mixing at low hydrodynamic stress and is well suited for the cultivation of suspended mammalian cells.

The different criteria to evaluate the risk of cell damage provided by Soos et al. [14], Werner et al. [13], and Li et al. [18] have been reconsidered in the present study. The tolerable maximum hydrodynamic stress and minimum Kolmogorov length scale provide different thresholds, and experimental studies [10,50] suggest that the hydrodynamic stress is the more relevant criterion for Chinese hamster ovary cells. Moreover, guided by the study of Li et al. [18], the average strain rate in the impeller zone is analyzed in both the impeller and the tank regions of the bioreactor. The values determined in the present study are higher than the optimal range reported by Li et al. [18] for Spodopterafrugiperda Sf9 and are not known for CHO and mammalian cells; therefore, a direct comparison for the same cell line is difficult. The different thresholds of tolerable levels for the three mentioned criteria for the evaluation of the risk of cell damage and the variations between cell lines indicate that further studies on the tolerance of the cultivated cells are required. It is proposed to also consider the extent of the volume and the time range during which cells are exposed to critical conditions, as cells are able to recover from sub-lethal conditions, which have not been investigated so far.

## Figures and Tables

**Table 1:** Operating conditions, * denotes the base case.

Condition	Working Volume *V*	Impeller Speed *n*	Sparging Rate *Q*	Sparger Type
#	[L]	[rpm]	[mL min${}^{-1}$]	
1	1.0	100	50	Microporous
2	2.4	100	50	Microporous
3	1.7	100	10	Microporous
4 *	1.7	100	50	Microporous
5	1.7	100	100	Microporous
6	1.7	50	50	Microporous
7	1.7	150	50	Microporous
8	1.7	100	50	Open pipe

## Data Availability

Data available on request due to restrictions related to intellectual property rights.

## References

[1] Nienow A.W. (2006). Reactor engineering in large scale animal cell culture. Cytotechnology.

[2] Li F., Vijayasankaran N., Shen A., Kiss R., Amanullah A. (2010). Cell culture processes for monoclonal antibody production. Monoclon. Antibodies.

[3] Sieck J.B., Cordes T., Budach W.E., Rhiel M.H., Suemeghy Z., Leist C., Villiger T.K., Morbidelli M., Soos M. (2013). Development of a Scale-Down Model of hydrodynamic stress to study the performance of an industrial CHO cell line under simulated production scale bioreactor conditions. J. Biotechnol..

[4] Davidson K.M., Sushil S., Eggleton C.D., Marten M.R. (2003). Using Computational Fluid Dynamics Software to Estimate Circulation Time Distributions in Bioreactors. Biotechnol. Prog..

[5] Sieblist C., Jenzsch M., Pohlscheidt M. (2016). Equipment characterization to mitigate risks during transfers of cell culture manufacturing processes. Cytotechnology.

[6] Villiger T.K., Morbidelli M., Soos M. (2015). Experimental determination of maximum effective hydrodynamic stress in multiphase flow using shear sensitive aggregates. AIChE J..

[7] Garcia-Ochoa F., Gomez E. (2009). Bioreactor scale-up and oxygen transfer rate in microbial processes: An overview. Biotechnol. Adv..

[8] Mainkowski M., Bodemeier S., Lübbert A., Bujalski W., Nienow A.W. (1994). Measurement of Gas and Liquid Flows in Stirred Tank Reactors with Multiple Agitators. Can. J. Chem. Eng..

[9] Laakkonen M., Moilanen P., Miettinen T., Saari K., Honkanen M., Saarenrinne P., Aittamaa J. (2005). Local Bubble Size Distributions in Agitated Vessel–Comparison of Three Experimental Techniques. Chem. Eng. Res. Des..

[10] Odeleye A.O., Marsh D.T., Osborne M.D., Lye G.J., Micheletti M. (2014). On the fluid dynamics of a laboratory scale single-use stirred bioreactor. Chem. Eng. Sci..

[11] Löffelholz C., Husemann U., Greller G., Meusel W., Kauling J., Ay P., Kraume M., Eibl R., Eibl D. (2013). Bioengineering Parameters for Single-Use Bioreactors: Overview and Evaluation of Suitable Methods. Chem. Ing. Tech..

[12] Kelly W.J. (2008). Using computational fluid dynamics to characterize and improve bioreactor performance. Biotechnol. Appl. Biochem..

[13] Werner S., Kaiser S., Krause M., Eibel D. (2014). CFD as a modern tool for engineering characterization of bioreactors. Pharm. Bioprocess..

[14] Soos M., Kaufmann R., Winteler R., Kroupa M., Lüthi B. (2013). Determination of maximum turbulent energy dissipation rate generated by a Rushton impeller through large eddy simulation. AIChE J..

[15] Kaiser S.C., Löffelholz C., Werner S., Eibl D., Minin I. (2011). CFD for Characterizing Standard and Single-use Stirred Cell Culture Bioreactors. Computational Fluid Dynamics Technologies and Applications.

[16] Seidel S., Maschke R.W., Werner S., Jossen V., Eibl D. (2021). Oxygen Mass Transfer in Biopharmaceutical Processes: Numerical and Experimental Approaches. Chem. Ing. Tech..

[17] Rathore A.S., Sharma C., Persad A.A. (2012). Use of computational fluid dynamics as a tool for establishing process design space for mixing in a bioreactor. Biotechnol. Prog..

[18] Li C., Teng X., Peng H., Yi X., Zhuang Y., Zhang S., Xia J. (2020). Novel scale-up strategy based on three-dimensional shear space for animal cell culture. Chem. Eng. Sci..

[19] Scully J., Considine L.B., Smith M.T., McAlea E., Jones N., O’Connell E., Madsen E., Power M., Mellors P., Crowley J. (2020). Beyond heuristics: CFD-based novel multiparameter scale-up for geometrically disparate bioreactors demonstrated at industrial 2kL-10kL scales. Biotechnol. Bioeng..

[20] Dreher T., Husemann U., Adams T., de Wilde D., Greller G. (2014). Design space definition for a stirred single-use bioreactor family from 50 to 2000 L scale. Eng. Life Sci..

[21] Kaiser S.C., Eibl R., Eibl D. (2011). Engineering characteristics of a single-use stirred bioreactor at bench-scale: The Mobius CellReady 3L bioreactor as a case study. Eng. Life Sci..

[22] Villiger T.K., Neunstoecklin B., Karst D.J., Lucas E., Stettler M., Broly H., Morbidelli M., Soos M. (2018). Experimental and CFD physical characterization of animal cell bioreactors: From micro- to production scale. Biochem. Eng. J..

[23] Muniz M., Sommerfeld M. (2020). On the force competition in bubble columns: A numerical study. Int. J. Multiph. Flow.

[24] Masterov M.V., Baltussen M.W., Kuipers J.A.M. (2018). Numerical simulation of a square bubble column using Detached Eddy Simulation and Euler–Lagrange approach. Int. J. Multiph. Flow.

[25] Xue J., Chen F., Yang N., Ge E. (2017). A Study of the Soft-Sphere Model in Eulerian- Lagrangian Simulation of Gas-Liquid Flow. Int. J. Chem. React. Eng..

[26] Wutz J., Lapin A., Siebler F., Schäfer J.E., Wucherpfennig T., Berger M., Takors R. (2016). Predictability of k_L_a in stirred tank reactors under multiple operating conditions using an Euler-Lagrange approach. Eng. Life Sci..

[27] Sungkorn R., Derksen J.J., Khinast J.G. (2011). Euler-Lagrange modeling of a gas-liquid stirred reactor with consideration of bubble breakage and coalescence. AIChE J..

[28] Sungkorn R., Derksen J.J., Khinast J.G. (2012). Modeling of aerated stirred tanks with shear-thinning power law liquids. Int. J. Heat Fluid Flow.

[29] Weber A., Bart H.J. (2018). Flow Simulation in a 2D Bubble Column with the Euler-Lagrange and Euler-Euler Method. Open Chem. Eng. J..

[30] Kreitmayer D., Gopireddy S., Matsuura T., Aki Y., Katayama Y., Nakano T., Eguchi T., Kakihara H., Nonaka K., Profitlich T. (2022). CFD-based and Experimental Hydrodynamic Characterization of the Single-Use Bioreactor Xcellerex^TM^ XDR-10. Bioengineering.

[31] Kreitmayer D., Gopireddy S.G., Matsuura T., Aki Y., Katayama Y., Kakihara H., Nonaka K., Profitlich T., Urbanetz N.A., Gutheil E. (2022). Numerical and experimental characterization of the single-use bioreactor Xcellerex^TM^ XDR-200. Biochem. Eng. J..

[32] Coroneo M., Montante G., Paglianti A., Magelli F. (2011). CFD prediction of fluid flow and mixing in stirred tanks: Numerical issues about the RANS simulations. Comput. Chem. Eng..

[33] Borys B.S., Roberts E.L., Le A., Kallos M.S. (2018). Scale-up of embryonic stem cell aggregate stirred suspension bioreactor culture enabled by computational fluid dynamics modeling. Biochem. Eng. J..

[34] Ferziger J.H., Peric M. (2002). Computational Methods for Fluid Dynamics.

[35] Versteeg H.K., Malalasekera W. (2007). An Introduction to CFD Finite Volume Method.

[36] Schiller L., Naumann A. (1935). A drag coefficient correlation. Zeit. Ver. Deutsch. Ing..

[37] Behzadi A., Issa R.I., Rusche H. (2004). Modelling of dispersed bubble and droplet flow at high phase fractions. Chem. Eng. Sci..

[38] Tomiyama A., Tamai H., Zun I., Hosokawa S. (2002). Transverse Migration of Single Bubbles in Simple Shear Flow. Chem. Eng. Sci..

[39] Kaiser S.C. (2014). Characterization and Optimization of Single-Use Bioreactors and Biopharmaceutical Production Processes Using Computational Fluid Dynamics. Ph.D. Thesis.

[40] Jamialahmadi M., Zehtaban M.R., Müller-Steinhagen H., Sarrafi A., Smith J.M. (2001). Study of Bubble Formation Under Constant Flow Conditions. Chem. Eng. Res. Des..

[41] Montante G., Horn D., Paglianti A. (2008). Gas-liquid flowand bubble size distribution in stirred tanks. Chem. Eng. Sci..

[42] Lamont J.C., Scott D.S. (1970). An Eddy Cell Model of Mass Transfer into the Surface of a Turbulent Liquid. AIChE J..

[43] Han P., Bartels D.M. (1996). Temperature Dependence of Oxygen Diffusion in H_2_O and D_2_O. J. Phys. Chem..

[44] OpenFOAM The OpenFOAM Foundation 2019. https://openfoam.org/news/funding-2019/.

[45] Brucato A., Ciofalo M., Grisafi F., Micale G. (1998). Numerical prediction of flow fields in baffled stirred vessels: A comparison of alternative modelling approaches. Chem. Eng. Sci..

[46] Kim T.K. (2015). T test as a parametric statistic. Korean J. Anesthesiol..

[47] Montante G., Paglianti A. (2015). Gas hold-up distribution and mixing time in gas–liquid stirred tanks. Chem. Eng. J..

[48] Minow B., Seidemann J., Tschoepe S., Gloeckner A., Neubauer P. (2014). Harmonization and characterization of different single-use bioreactors adopting a new sparger design. Eng. Life Sci..

[49] Gelves R., Dietrich A., Takors R. (2014). Modeling of gas–liquid mass transfer in a stirred tank bioreactor agitated by a Rushton turbine or a new pitched blade impeller. Bioprocess Biosyst. Eng..

[50] Neunstoecklin B., Stettler M., Solacroup T., Broly H., Morbidelli M., Soos M. (2015). Determination of the maximum operating range of hydrodynamic stress in mammalian cell culture. J. Biotechnol..

[51] Haringa C., Noorman H.J., Mudde R.F. (2017). Lagrangian modeling of hydrodynamic–kinetic interactions in (bio)chemical reactors: Practical implementation and setup guidelines. Chem. Eng. Sci..

